# Entomological Surveillance and Cantharidin Concentrations in *Mylabris variabilis* and *Epicauta rufidorsum* Blister Beetles in Slovenia

**DOI:** 10.3390/ani11010220

**Published:** 2021-01-18

**Authors:** Breda Jakovac-Strajn, Diana Brozić, Gabrijela Tavčar-Kalcher, Janja Babič, Tomi Trilar, Modest Vengust

**Affiliations:** 1 Veterinary Faculty, University of Ljubljana, 1000 Ljubljana, Slovenia; breda.jakovacstrajn@vf.uni-lj.si (B.J.-S.); gabrijela.tavcar-kalcher@vf.uni-lj.si (G.T.-K.); janja.babic@vf.uni-lj.si (J.B.); 2 Department of Animal Nutrition and Dietetics, Faculty of Veterinary Medicine, University of Zagreb, Heinzelova 55, 10000 Zagreb, Croatia; diana.brozic@vef.hr; 3 Slovenian Museum of Natural History, 1000 Ljubljana, Slovenia; ttrilar@pms-lj.si

**Keywords:** cantharidin toxicosis, LC-MS/MS, blister beetle, meloidae, forage contamination, horse

## Abstract

**Simple Summary:**

True blister beetles (genus *Epicauta*, family Meloidae) produce cantharidin, which is a potent blistering agent. Cantharidin toxicosis is well documented in humans and animals. Drought and modern harvesting techniques are thought to increase the likelihood of blister beetle contamination of forage and cantharidin intoxication in animals. Local presence, behavioral patterns, and cantharidin concentration were studied in *Mylabris variabilis* and *Epicauta rufidorsum*. In this study, *E. rufidorsum* was found to be the most likely source of forage contamination due to its local abundance, swarming activity, and tendency to reside in the green parts of plants after cutting. Cantharidin was detected in the bodies of both blister beetles species studied. It is likely that modern forage harvesting methods, which involve simultaneous processing of forage after cutting, increase the incidence of cantharidin toxicoses in animals. Delaying these processes by a few minutes would reduce the likelihood of *Epicauta* contamination of the forage.

**Abstract:**

True blister beetles (genus *Epicauta*, family Meloidae) produce cantharidin, which can cause toxicosis in humans and animals. Some recent reports suggest that poisoning by the blister beetle has occurred in the Mediterranean part of Slovenia, which has never been reported before. Drought and modern harvesting techniques are thought to increase the likelihood of blister beetle forage contamination and cantharidin intoxication in animals. A survey of fields associated with blister beetle contamination was conducted and the Meloid species present were identified. Entomological surveillance was conducted for *Mylabris variabilis* and *Epicauta rufidorsum*. Cantharidin concentrations were also measured in both blister beetle species. Cantharidin concentration in *Mylabris variabilis* (*n* = 17) ranged from 0.038 to 0.354 µg/mg (mean 0.151 µg/mg). Cantharidin concentration in *Epicauta rufidorsum* (*n* = 36) ranged from 0.055 to 0.341 µg/mg (mean 0.142 µg/mg). Both species exhibited variable concentrations of cantharidin that could not be associated with their biology, sex, age, size, and/or reproductive status. *Epicauta rufidorsum* have never previously been studied as a possible source of forage contamination, nor have cantharidin concentrations been determined in this species. It is the most likely source of forage contamination due to its abundance in the investigated fields, its swarming activity, and its tendency to reside in the green parts of plants immediately after cutting. Delaying the simultaneous processing and storage of forage after cutting would reduce the likelihood of forage contamination by blister beetles, as they can then retreat to the ground or fly away.

## 1. Introduction

True blister beetles (genus *Epicauta*, family Meloidae) [1] are known for their ability to produce a vesicant chemical, the monoterpene anhydride compound cantharidin, which is a potent blistering agent [2,3]. The main function of cantharidin is to protect the eggs of the insects from potential predators [4,5,6]. The production of cantharidin varies greatly depending on species, size of the beetle, sex, season, and food source [7,8].

Cantharidin toxicosis is well documented in humans, birds, horses, and other mammals [9,10,11,12,13,14,15]. It is most frequently reported in horses in North America and is related to consumption of alfalfa hay contaminated with *Epicauta* beetles [9,16,17,18,19]. Cantharidin toxicosis in humans is often associated with cases where cantharidin is used as a part of traditional remedies for its alleged medical and/or aphrodisiac purposes [13,20]. Ingestion of toxic doses of cantharidin has been reported to affect multiple organ systems. The pathological changes are related to direct inhibition of protein phosphatase 2A receptors in all tissues, which regulate a variety of vital cellular functions [21]. Epithelial tissues have a rapid turnover rate and are associated with early onset of disease; therefore, clinical signs are most commonly associated with gastrointestinal and urogenital tract disorders. Sudden death is possible [3,19]. Cantharidin is a stable toxin, which does not degrade significantly by storage or forage processing [3].

Possible cantharidin toxicoses were reported in a small number of horses by local veterinarians in the Mediterranean part of Slovenia between the years 2010–2013. The reports included observations of insect parts in stomach contents collected by nasogastric intubation, which probably belonged to beetles that were also found in freshly cut grass fed to the horses. The horses were not sent for necropsy, and the beetles were not preserved or further categorized to a specific species. Food poisoning caused by insects would be an extremely unusual event in the region; therefore, blister beetle poisoning was not considered as a possible culprit until several weeks later when an equine specialist was consulted.

The concentration of cantharidin in Meloid beetles in the northern Mediterranean area has already been studied and appears to be similar to that in regions where the disease is more endemic [8]. However, Meloid beetles have never been studied as a potential forage contaminant in the region. Therefore, the objectives of this study were (1) to survey pastures in the affected region for the presence of Meloid beetles, (2) to conduct an observational sampling of blister beetles, and (3) to determine cantharidin concentrations in beetles that are the most likely source of poisoning.

## 2. Materials and Methods

### 2.1. Entomological Surveillance

Entomological observations were carried out in the Mediterranean part of Slovenia for three years (2013–2016) in June, July, and August. Locations investigated were consistent with previous reports of possible blister beetle intoxication in horses. Coordinates of the investigated area were: 45.575056° N 13.907306° E; 45.613444° N 13.907583° E; 45.525750° N 13.691001° E; and 45.524944° N 13.687056° E.

The basic biology of several *Epicauta* species was extrapolated from the Database of Invertebrate Pictures of the Slovenian Museum of Natural History [22] and the Global Biodiversity Information Facility [23]. Based on this, *Mylabris variabilis* [24] and *Epicauta rufidorsum* [25] were selected for this study due to their geographical distribution, evident abundance on the fields investigated, and their distinctly different behavior patterns with regard to occupying/roaming in their environment. *Epicauta rufidorsum* has not yet been investigated as a potential contaminant of forage. Changes in the natural environment of the blister beetles were made by causing disturbances through our physical presence (direct hunting of individuals and observation of their behavior), by cutting/ripping vegetation where blister beetles were present and then observing their activity using a combination of focal-animal and point sampling methods [26].

The sampled beetles were stored in a plastic box and placed in the freezer (−16 °C). The identification of the insects, including sex differentiation, was performed at the Slovenian Museum of Natural History. The insects were then analyzed for the presence of cantharidin in their bodies, and differences between species and between genders within species were analyzed.

### 2.2. Cantharidin Detection

#### 2.2.1. Standards and Chemicals

Cantharidin standard (certified purity 99.8%) was purchased from Sigma-Aldrich (Steinheim, Germany). Stock standard solution and the working standard solutions were prepared in the methanol-water mixture (30 + 70) and stored in a refrigerator. Methanol (Honeywell, Seelze, Germany), chloroform, hydrochloric acid, and formic acid (Merck, Darmstadt, Germany) were of analytical or chromatography-grade purity. Deionized water was prepared using a Milli-Q system (Millipore, Bedford, MA, USA).

#### 2.2.2. Analytical Procedure

Sample preparation and cantharidin determination were based on analytical procedures described elsewhere [27,28]. All beetles were put into 7 mL glass vials separately, weighed, and freeze-dried for 24 h at −80 °C and 0.01 mbar using an ALPHA 2–4 LSC freeze-dryer (Christ, Osterode am Harz, Germany). The dried bodies were weighed again. To each tube, 800 μL of 6M hydrochloric acid was added and the mixture was boiled for 30 min. To the dissolved (digested) samples, 400 μL of chloroform was added, shaken on a Vortex mixer, and centrifuged for 5 min at 3500 rpm. The organic phase was transferred to vials and evaporated under vacuum to dryness using a Syncore Polyvap system (Büchi, Flawil, Switzerland). The dry residue was reconstituted in 400 μL of methanol-water mixture (30 + 70). An aliquot of the solution (10 μL) was injected into the UPLC-MS/MS system (Acquity UPLC H Class system) coupled with a triple-quadrupole mass spectrometer (Xevo TQ MS) equipped with an electrospray ionization (ESI) interface and MassLynx software for data collection and processing (Waters, Milford, MA, USA). The vials were kept in the autosampler at 10 °C.

#### 2.2.3. LC-MS/MS Analysis

Chromatographic separations were performed on a Zorbax Eclipse Plus C18 Rapid Resolution HD column, 2.1 × 100 mm, 1.8 µm (Agilent Technologies, Santa Clara, CA, USA). The chromatographic separation was performed by mixing of two mobile phase components (A and B) in an isocratic mode in the ratio 70:30. Mobile phase component A was deionized water and component B was methanol, both containing 0.1% formic acid. Sample volume 10 µL was injected into the column with a mobile phase flow rate 0.5 mL/min and temperature 45 °C. MS/MS analysis was carried out using ESI in positive ionization mode and the triple-quadrupole mass spectrometer operated in multiple reactions monitoring (MRM) mode in following conditions: source temperature 150 °C, desolvation temperature 500 °C, the capillary voltage 1.5 kV, the cone gas flow 20 L/h, and the desolvation gas flow 850 L/h. For measuring the presence of cantharidin, the transitions 197.0 > 94.99 and 197.0 > 123.0 were monitored. The quantification was done using matrix-matched calibration. The concentration of cantharidin was expressed to the dry weight of beetles.

#### 2.2.4. Statistical Analysis

Data were analyzed using GraphPadPrism6 software [29] between the groups using the unpaired *t*-test (parametric) or Mann–Whitney test (non-parametric unpaired *t*-test) if data failed to pass the Shapiro–Wilk normality test (*p* < 0.05). To assess the relationship between the mass of the beetle and cantharidin content, Spearman’s rank correlations were conducted. A value of *p* < 0.05 was used to determine statistical significance. Descriptive statistics are reported in the text as mean ± standard deviation (SD).

## 3. Results

### 3.1. Entomological Observations

Several field observations were made over a 3-year period during the summer months. *Mylabris variabilis* and *E. rufidorsum* were identified as common Meloid species in the fields associated with reports of blister beetle infestations. No other Meloid species were identified.

*Mylabris variabilis* is a common species distributed throughout most of southern Europe, from the Iberian Peninsula to southern Russia, Caucasus, Transcaucasia, Near East and northern Levant, Middle East, Siberia, and central Asia [24,30]. We have detected the beetle in the environment during the hot summer months (July, August). Our observations did not document any significant swarming or congregating behavior of this species. They usually fly from plant to plant and are rarely seen on the ground. The adults feed on flowers [31]. Mating is mostly individual and occurs on plants. These beetles have cantharidin in their body as reported herein and other studies [8]. However, due to their biology, especially their mobility, they do not seem to pose a risk for contamination of forage. They can escape field management such as mowing and crop storage and migrate from drought-affected areas.

*Epicauta rufidorsum* is distributed in France, Italy, Austria, Hungary [24], Slovenia [22], Bulgaria, Turkey, Caucasus, South Russia [31,32,33,34], Uzbekistan, and Kazakhstan [35]. Unlike *M. variabilis*, it is a less investigated blister beetle with poorly understood biology. It was previously identified as a possible pest of potatoes and sunflowers, which also indicates swarming behaviour [36,37]. In Slovenia, they are found on xeric meadows, where they feed mainly on wild leguminous plants. Swarms are formed during mating, with several beetles concentrated on one plant. During the summer drought, they were observed congregating on irrigated football turfs, which had not been observed before. In contrast to *M. variabilis*, they rarely fly. They mostly dwell on the ground. When they are alarmed, they first try to hide in the low vegetation or debris of the ground. If the hiding is unsuccessful and the disturbance persists, they start climbing plants vertically, and when they reach the top or end of the plant, they fly away from the danger zone. The same can be observed when mowing grass or crops: they start climbing vertically on fallen plants to reach the top. This behavior pattern causes a high concentration of beetles on green parts of plants shortly after cutting. When the disturbance is over, they fly away or move back to the ground.

### 3.2. Total Cantharidin Concentrations

**Figure 1:**
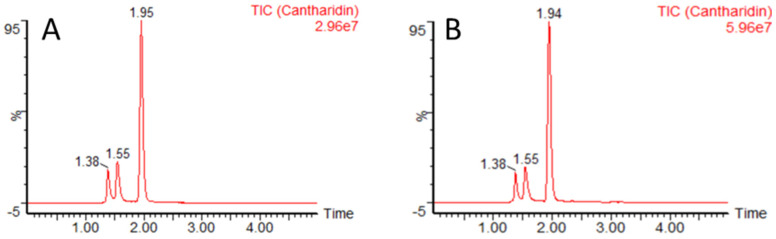
Chromatogram of cantharidin in blister beetle in the sample, spiked at the limit of quantification (LOQ) of 0.6 ng/mg (**A**) and in the positive beetle sample of *Mylabris variabilis* (concentration was above or equal to LOQ) (**B**). The retention time of cantharidin was at 1.95 min.

The concentration of cantharidin in 53 blister beetle samples was determined by LC-MS/MS. Cantharidin was determined in 17 *M. variabilis* (7 males and 10 females) and in 36 *E. rufidorsum* (33 males and 3 females) blister beetles. Samples containing cantharidin at concentrations equal to or above the limit of quantification (LOQ) of 0.6 ng/mg were considered positive. The chromatogram of cantharidin in the sample spiked at LOQ and, for example, the positive sample from *M. variabilis* is shown in Figure 1A,B, respectively.

The total occurrence of cantharidin in both blister beetle species is shown in Table 1. Cantharidin was determined in all 53 blister beetles. The mass of cantharidin determined in the individual blister beetles ranged from 6.5 µg to 51.8 µg with a median of 21.4 µg. The concentration of cantharidin in blister beetles ranged from 0.038 µg/mg to 0.354 µg/mg per wet body weight, and 0.100 µg/mg to 1.08 µg/mg dry body weight. The average wet and dry body weights of the blister beetles were 0.166 g and 0.064 g, respectively.

### 3.3. Cantharidin Concentrations in M. variabilis and E. rufidorsum

**Figure 2:**
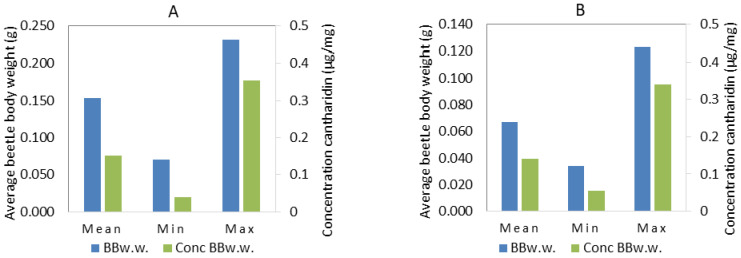
Graphical discriptive statistics for blister beetle wet body weight (BBw.w., blue bars) and concentration of cantharidin per Bw.w. (Conc BBw.w., green bar) in blister beetles: (**A**) *M. variabilis* (*n* = 17) and (**B**) *Epicauta rufidorsum* (*n* = 36). Mean: average value; Min: minimum value, Max: maximum value.

Cantharidin concentration was different in both species (Figure 2A,B). In 17 samples of *M. variabilis*, the concentration of cantharidin ranged from 0.038 µg/mg to 0.354 µg/mg of their wet weight, with an average concentration of 0.151 µg/mg. The wet body weight of *M. variabilis* ranged from 0.070 g to 0.231 g. The average wet body weight was 0.154 g. In 36 samples of *E. rufidorsum*, the concentration of cantharidin ranged from 0.055 µg/mg to 0.341 µg/mg of their wet weight, with an average concentration of 0.142 µg/mg. The wet body weight in *E. rufidorsum* ranged from 0.080 g to 0.334 g. The average wet body weight was 0.172 g.

Cantharidin concentration also varied between individuals in both species, as shown in Table 2. In *M. variabilis*, body wet weight ranged from 0.120 g to 0.223 g in males, while in females it ranged from 0.070 g to 0.204 g. The body wet weight in *E. rufidorsum* ranged from 0.080 g to 0.334 g in males, and from 0.115 g to 0.230 g in females (Table 2).

When dry matter concentrations were compared, no significant differences in mean cantharidin content were found between species *M. variabilis* and *E. rufidorsum* (*p* = 0.164). However, there was evidence of different body weight between the two investigated species (*p* = 0.049), with *E. rufidorsum* having an average dry weight of 0.067 ± 0.020 g and *M. variabilis* of 0.056 ± 0.017 g.

## 4. Discussion

Several studies have identified blister beetles as a potential source of cantharidin poisoning in horses, other animals, and humans. Most of the cantharidin toxicosis studies are from North America, where drought and modern harvesting techniques have been linked to the introduction of *Epicauta* beetles into animal feed [17]. In this study, *E. rufidorsum* was identified as a possible source for forage contamination in the Mediterranean part of Slovenia. *Epicauta rufidorsum* was abundant in the investigated fields and showed a consistent tendency to climb vertically into the stem and leaves when disturbed. Fresh cut grass feeding and/or the immediate crimping and storage/preservation of the forage after cutting, which is becoming a predominant harvesting technique in the region, increase the likelihood of *E. rufidorsum* being present in the forage. Traditional harvesting techniques such as hay or haylage production reduce the possibility of blister beetle contamination of the forage, as the beetles have time to disperse. Meloid beetle species with behavior patterns like *M. variabilis* are less likely to contaminate the forage.

Cantharidin concentrations vary widely between and within Meloid species as also determined in this study. In various studies, concentrations of less than 0.04 to up to 30.3 mg of cantharidin per individual beetles have been found [2,38]. Gisondi et al. [8] examined the cantharidin concentration of *M. variabilis* collected in central Italy and reported the range between 0.001 and 0.994 mg per beetle, with the highest concentrations being significantly higher than cantharidin content found in this study. Nikbakhtzadeh and Tirgari [39] reported similar results for the same species collected in Hamedan province, Iran. Species from the genus *Epicauta* identified in Kansas, USA also contained highly variable concentrations of cantharidin [40]. In *Berberomeloe majalis* from central Spain, reported levels in adult beetles varied from 0.035 to 109.2 mg [41,42,43]. Males of Meloidae are known to contain higher concentrations of cantharidin than females [39,44,45]. Our results showed no sex-specific differences in cantharidin concentration, which was also reported regionally by Gisondi et al. [8]. The absence of a significant difference between males and females can be attributed to the transfer of cantharidin from males to females during copulation [8,27,46,47]. The cantharidin concentrations in this and other studies should, therefore, be attributed to a well-known phenomenon of variation in titers of arthropod chemical defense depending on their biology, sex, age, size, and reproductive status [8,40,44,48].

Based on our investigation, *M. variabilis* is an unlikely source of forage contamination. However, the abundance of *E. rufidorsum* on investigated fields and its hazard evasive behavior patterns are of concern. To date, no specific biology or information on cantharidin concentrations of *E. rufidorsum* has been reported in the literature. They are more difficult to observe, identify, and collect than some other Meloid beetle species, which is likely the reason for the lack of critical observations of this species in their habitat. It is important to note that the mass of *E. rufidorsum* was 16% higher than that of *M. variabilis*. This could also be the reason why *E. rufidorsum* are bound to the ground and rarely fly. Cantharidin concentration has been reported to be linearly and positively related to the dry weight of individual beetles [49]. Therefore, fewer *E. rufidorsum* individuals would be required for relevant cantharidin contamination of the forage compared to *M. variabilis*.

## 5. Conclusions

*Epicauta rufidorsum* was the most ubiquitous and most common blister beetle species found in the fields which were associated with reported forage contamination. Reports of forage contamination by blister beetles have only recently been observed. It appears that repeated drought events in the region in modern times [50] may have influenced the behavior of Meloid beetles. Current climate change will potentiate meteorological and hydrological drought events [50] and increase the possibility of cantharidin toxicosis in animals in the region. The information gathered in this study may provide veterinarians, horse owners, and livestock farmers with tools to prevent clinical cantharidin intoxications in their animals.

## Figures and Tables

**Table 1:** Cantharidin in blister beetles analyzed with LC-MS/MS.

Descriptive Statistics	m_Canth._ (µg)	BBd.w. (g)	Conc. d.w. (µg/mg)	BBw.w. (g)	Conc. w.w. (µg/mg)
Mean	22.2	0.064	0.377	0.166	0.145
Min	6.5	0.026	0.100	0.070	0.038
Max	51.8	0.123	1.08	0.334	0.354
Median	21.4	0.065	0.322	0.165	0.128

Legend: m_Canth_: cantharidin mass in beetle; BB: blister beetle; w.w.: wet weight; d.w.: dry weight; Conc.: concentration of cantharidin.

**Table 2:** Body weight and cantharidin concentration in *Mylabris variabilis* and *Epicauta rufidorsum.*

Species	Sex	Body w.w. (g)	Body d.w. (g)	Cantharidin per Beetle (mg)	Cantharidin Concentration d.w. (%)
*M. variabilis*	M (*n* = 7)	0.172 ± 0.042	0.067 ± 0.015	0.022 ± 0.012	0.032 ± 0.014
	F (*n* = 10)	0.141 ± 0.046	0.049 ± 0.016	0.022 ± 0.009	0.049 ± 0.026
	Total	0.154 ± 0.046	0.056 ± 0.017	0.022 ± 0.010	0.042 ± 0.023
	^1^*p* value M × F	0.281	0.066	0.944	0.239
*E. rufidorsum*	M (*n* = 33)	0.173 ± 0.054	0.067 ± 0.019	0.022 ± 0.006	0.036 ± 0.015
	F (*n* = 3)	0.164 ± 0.060	0.070 ± 0.027	0.025 ± 0.009	0.037 ± 0.001
	Total	0.172 ± 0.054	0.067 ± 0.020	0.022 ± 0.006	0.036 ± 0.014
	^1^*p* value M × F	0.628	0.990	0.405	0.238

Values are mean ± SD; wet weight, w.w.; dry weight, d.w.; ^1^ *p* value is determined by unpaired *t*-test (parametric) or Mann–Whitney test (non-parametric); male, M; female, F.

## Data Availability

The datasets used and/or analyzed during the current study are available from the corresponding author on reasonable request.
